# BRMS1L promotes chemotherapy sensitivity by inhibiting autophagy in breast cancer

**DOI:** 10.3389/fgene.2025.1670001

**Published:** 2025-11-07

**Authors:** Yuan Li, Dian Zhang, Junhao Zhao, Mei Yang, Yiping Wang, Peiyao Lee, Shaohua Qu

**Affiliations:** 1 Department of Breast Surgery, The First Affiliated Hospital of Jinan University, Jinan University, Guangzhou, China; 2 Department of Breast Surgery, JiangMen Maternity and Child Healthcare Hospital, Jiangmen, China

**Keywords:** BRMS1L, ATG5, autophagy, chemotherapy sensitivity, breast cancer

## Abstract

Chemoresistance remains a crucial obstacle in breast cancer therapy. The mechanisms underlying chemoresistance need to be explored urgently and in depth. Breast cancer metastasis suppressor 1 like (BRMS1L), a core component of the Sin3A–histone deacetylase (HDAC) co-repressor complex, has been reported to suppress breast cancer metastasis through epigenetically regulating the Wnt signal pathway. However, whether BRMS1L could regulate chemosensitivity has not been explored. Herein, we found that higher BRMS1L expression was significantly correlated with increased chemotherapy sensitivity and better prognosis in patients receiving neoadjuvant chemotherapy. *In vitro* experiments confirmed that chemoresistant breast cancer cells exhibited decreased BRMS1L expression compared to chemosensitive cells. *In vivo* experiments in nude mice demonstrated that BRMS1L markedly strengthened the chemotherapy effects on xenografts. RNA sequencing (RNA-seq) was performed to elucidate the molecular mechanism underlying BRMS1L-mediated chemosensitivity. Bioinformatics analysis indicated that BRMS1L promotes chemotherapy sensitivity by regulating cellular autophagy. Furthermore, chemoresistant breast cancer cells exhibited elevated autophagy levels, and ectopic expression of BRMS1L significantly suppressed protective autophagy through downregulating ATG5. Collectively, these results revealed that BRMS1L enhances chemotherapy sensitivity via inhibiting protective autophagy. To our knowledge, this is the first study that showed that reduced BRMS1L expression is associated with poor response to neoadjuvant chemotherapy and unfavorable prognosis in breast cancer patients. Our findings reveal a novel role of BRMS1L in chemosensitivity and highlight its potential clinical application in the treatment of breast cancer.

## Introduction

Breast cancer is one of the most common malignancies with increasing incidence in women and the leading cause of female cancer death worldwide. Due to the rapid development of novel chemotherapy drugs and target regimens, the prognosis of breast cancer patients has been greatly improved. However, the mortality shows less improvement in the past decades (Siegel et al., 2023). Among the current therapeutic strategies, chemotherapy remains the main treatment approach in clinical practice. However, chemotherapy resistance unavoidably results in treatment failure and poor prognosis in breast cancer patients. Therefore, exploration of the mechanisms underlying chemotherapy resistance will contribute to more effective treatment and thus improve patient survival.

At present, the mechanisms of chemotherapy resistance vary, including increased ABC transporter expression, cancer stem cells, metabolic alterations, impairment of DNA damage repair mechanisms, changes in the tumor microenvironment, autophagy-mediated drug resistance, and other gene mutations and epigenetic changes (Sun et al., 2022; Qin et al., 2018; Liu et al., 2021; Ferrari et al., 2022; Liu et al., 2022; Mentoor et al., 2018). Among these mechanisms, autophagy-mediated chemotherapy resistance has gained more and more attention (Cuomo et al., 2019; Cocco et al., 2020; Zamame Ramirez et al., 2021; Jung et al., 2020; Hu et al., 2021; Yu et al., 2022; Lin et al., 2022; Oh et al., 2024; An et al., 2021). Autophagy is a conservative lysosomal degradation pathway for the removal of cytoplasmic components to maintain cellular homeostasis (Cuomo et al., 2019). During the cancer development and progression, autophagy plays a double-edged sword role (Cocco et al., 2020; Zamame Ramirez et al., 2021; Jung et al., 2020). On the one hand, it could promote tumor cell death and thereby function as a tumor suppressor when tumor originates (Cocco et al., 2020). On the other hand, autophagy can function as a cytoprotective mechanism that promotes tumor cell survival under stress conditions such as hypoxia and nutrient starvation (Zamame Ramirez et al., 2021; Jung et al., 2020). Numerous studies identified that autophagy is one of the stress-protective mechanisms and its activation has been proven to induce chemoresistance (Hu et al., 2021; Yu et al., 2022; Lin et al., 2022; Oh et al., 2024). Cancer cells must activate their self-protective mechanisms to survive the stressful conditions induced by drug treatment, which suggests that they can evade chemotherapy drug-induced apoptosis via enhanced autophagy levels, subsequently leading to chemotherapy resistance (An et al., 2021). Thus, specific interventions that block autophagy have been considered a novel therapeutic strategy to enhance chemosensitivity and improve the prognosis of breast cancer patients (Hashemi et al., 2023).

Breast cancer metastasis suppressor 1 like (BRMS1L) is a component purified from the Sin3–histone deacetylase (HDAC) complex, which is capable of histone deacetylation and transcription suppression (Nikolaev et al., 2004). A previous study revealed that BRMS1L suppresses breast cancer invasiveness and metastasis by inhibiting epithelial–mesenchymal transition (EMT) (Gong et al., 2014). BRMS1L silences *FZD10* by recruiting HDAC1 to its promoter, leading to H3K9 deacetylation and suppression of aberrant WNT3-FZD10-β-catenin signaling. However, it remains obscure whether BRMS1L is associated with autophagy via regulating chemosensitivity of breast cancer cells.

In the present study, we found that reduced BRMS1L expression correlates with poor response to neoadjuvant chemotherapy and unfavorable prognosis in breast cancer patients. Chemoresistant breast cancer cells exhibited elevated autophagy levels, which is a mechanism that helps the cells survive and resist the drug. Additionally, chemoresistant breast cancer cells exhibited decreased BRMS1L expression. RNA-sequencing analysis of chemoresistant breast cancer cells indicated that autophagy plays a specific role in driving chemotherapy resistance. Furthermore, BRMS1L significantly enhanced chemotherapy sensitivity by inhibiting protective autophagy in breast cancer cells. *In vivo* experiments further validated that BRMS1L exerts potent antitumor effects, highlighting its potential clinical application in the treatment of breast cancer.

## Results

### High BRMS1L expression correlates with an improved response to neoadjuvant chemotherapy (NACT) and better prognosis

**FIGURE 1:**
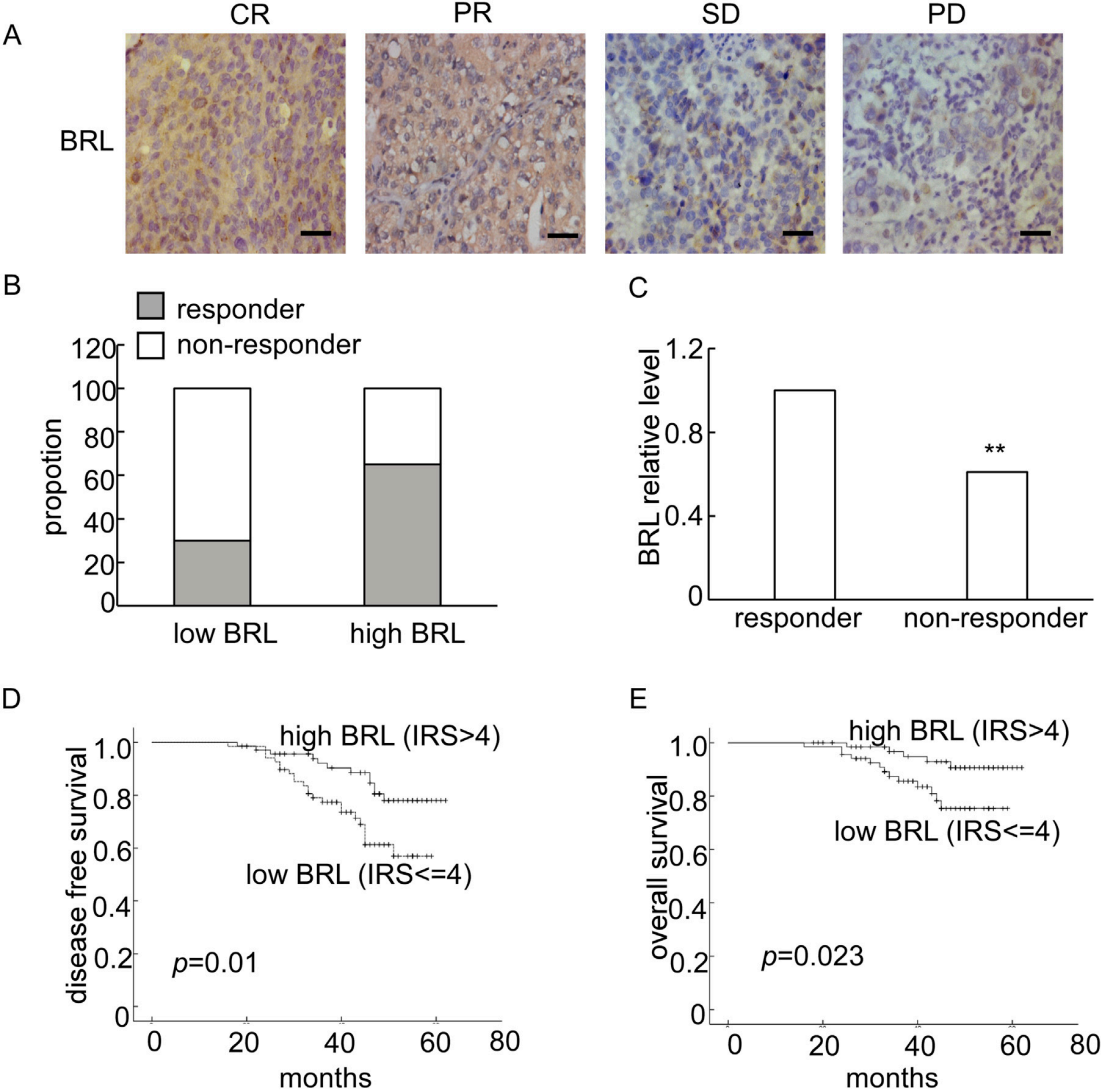
The expression of BRMS1L was correlated with neoadjuvant chemotherapy (NACT) response and prognosis of breast cancer patients. **(A)** Representative IHC of BRMS1L in breast tissues with different response to NACT. Scale bar corresponds to 50 mm. CR, complete response; PR, partial response; SD, stable disease; PD, progressed disease. **(B)** Percentage of pCR in patients stratified by BRMS1L expression. **(C)** The BRMS1L mRNA expression was measured using qRT-PCR in breast cancer tissues. **(D, E)** The Kaplan–Meier disease-free survival (DFS) and overall survival (OS) curves of patients with low (IR S≤ 4) and high (IRS > 4) BRMS1L levels, with a median follow-up period of 45 months. BRL, BRMS1L. Non-responders vs. responders, **p < 0.01.

BRMS1L has been identified as a breast cancer metastasis suppressor, but its clinical relevance with NACT remains unclear. In the present study, 138 breast cancer tissue samples from patients who received neoadjuvant chemotherapy (NACT) were collected. To examine whether the BRMS1L expression is associated with the efficacy of NACT, immunohistochemistry (IHC) for BRMS1L protein expression was first performed. The results revealed that patients with high BRMS1L expression (IRS > 4) significantly correlated with an elevated response to chemotherapy (CR + PR), whereas the low BRMS1L level correlated with a poor response to chemotherapy (SD + PD, Figures 1A,B). Similarly, the BRMS1L mRNA level, determined through quantitative real-time polymerase chain reaction (qRT-PCR), was much higher in responders than in non-responders (fold change 1.88, Figure 1C).

Furthermore, we analyzed the association between BRMS1L expression and the clinicopathological status of 138 patients with breast cancer. It revealed that high BRMS1L expression was associated with a smaller tumor size, lower grade, less lymph node involvement, and lower relapse rate. However, there was no correlation between BRMS1L and patient age, hormone receptor (HR), or HER-2 status (Table 1). Among the 138 patients, there were 83 chemotherapy-sensitive patients and 55 chemotherapy-resistant patients. According to the statistical analysis, patients with high BRMS1L expression (IRS > 4) were more sensitive to chemotherapy, but patients with low BRMS1L levels (IRS ≤ 4) are more resistant to chemotherapy (Supplementary Table S1). Moreover, to determine the correlation between BRMS1L expressions and treatment paradigms, we found that there was no correlation between BRMS1L and NACT regimens, surgical therapy, number of chemotherapy cycles, or endocrine therapy (Supplementary Table S2). In addition, the Kaplan–Meier survival curve with a median follow-up of 45 months demonstrated that patients with high BRMS1L expression had a better disease-free survival and a better overall survival than those with low BRMS1L expression (Figures 1D,E). Taken together, these data suggest that high BRMS1L expression correlates with high chemotherapy sensitivity and better prognosis.

**TABLE 1:** Association between BRMS1L expression and clinicopathologic features of 138 breast cancer patients [No (%)].

Item	Total no.	BRMS1L expression			*p*
		High (IRS > 4)	Low (IRS ≤ 4)		
Age (years)					
≤50	63	29	34	1.021	0.393
>50	75	41	34		
Tumor size					
2–5 cm	78	46	32	4.885	0.039
>5 cm	60	24	36		
Grade					
I	23	17	6	6.918	0.031
II	55	28	27		
III	60	25	35		
Lymph node status					
N0	18	8	10	11.228	0.011
N1	45	32	13		
N2	53	21	32		
N3	22	9	13		
HR status					
Positive	81	45	36	1.831	0.226
Negative	57	25	32		
HER-2 status					
Negative	89	40	49	3.351	0.077
Positive	49	30	19		
Relapse					
No	103	58	45	5.07	0.024
Yes	35	12	23		

The χ^2^ test was used to calculate p-values.

### BRMS1L promotes chemotherapy sensitivity in breast cancer cells

**FIGURE 2:**
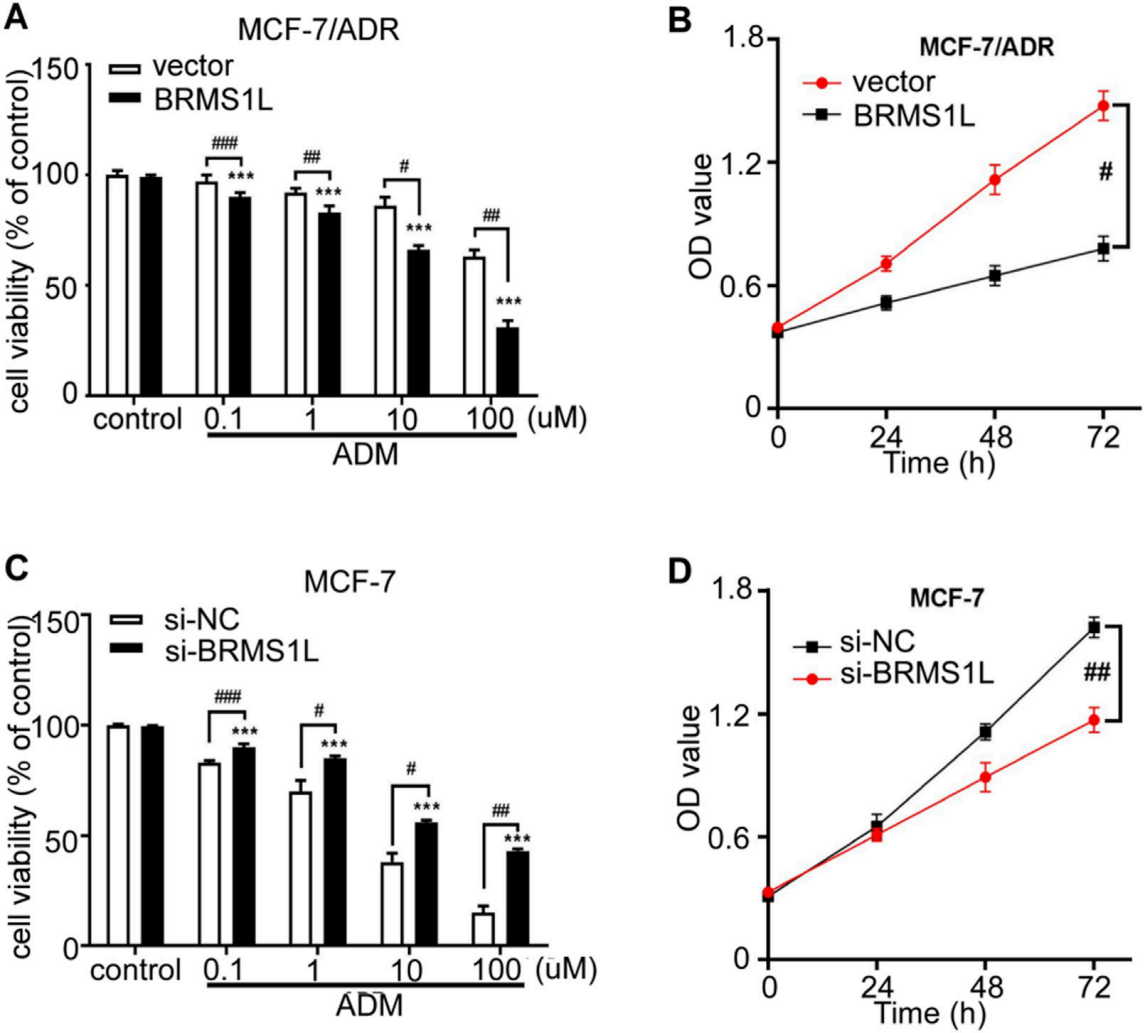
BRMS1L promotes chemosensitivity in breast cancer cells. **(A, C)** Cell viability was determined under treatment of different concentrations of ADM in MCF-7/ADR and MCF-7 cells. **(B, D)** Cell viability was determined after 24 h, 48 h, or 72 h in MCF-7/ADR and MCF-7 cells using CCK-8 assays. ADM vs. control; ***, *p* < 0.001. BRMS1L vs. vector, ###, *p* < 0.001; ##, *p* < 0.01; #, *p* < 0.05.

Adriamycin (ADM) is widely used to treat breast cancer patients with a high risk of recurrence and plays a vital role as one of the standard treatments in breast cancer for a long time. However, the efficacy of chemotherapy varies due to primary resistance to ADM. In this study, to investigate the effect of BRMS1L on cell viability to chemotherapy, we treated MCF-7 and Adriamycin-resistant MCF-7/ADR cells with a series of ADM concentrations: 0.1 μM, 1 μM, 10 μM, and 100 μM. Cell viability was determined using the Cell Counting Kit-8 (CCK-8) assay after 48 h of ADM treatment. The results showed that the ectopic expression of BRMS1L significantly reduced the cell viability of MCF-7/ADR cells in a dose-dependent manner compared to control cells. In addition, we determined the cell viabilities treated with 10 μM ADM following 24 h, 48 h, and 72 h. The CCK-8 assays revealed that the ectopic expression of BRMS1L reduced the cell viability of MCF-7/ADR cells in a time-dependent manner compared to MCF-7 cells (Figures 2A,B). On the contrary, silencing BRMS1L significantly enhanced ADM cytotoxicity in MCF-7 cells compared with the control group (Figures 2C,D; Supplementary Figure S1). Collectively, these data suggest that BRMS1L enhances chemotherapy sensitivity of breast cancer cells.

### BRMS1L inhibits ADM-induced autophagy in breast cancer cells

**FIGURE 3:**
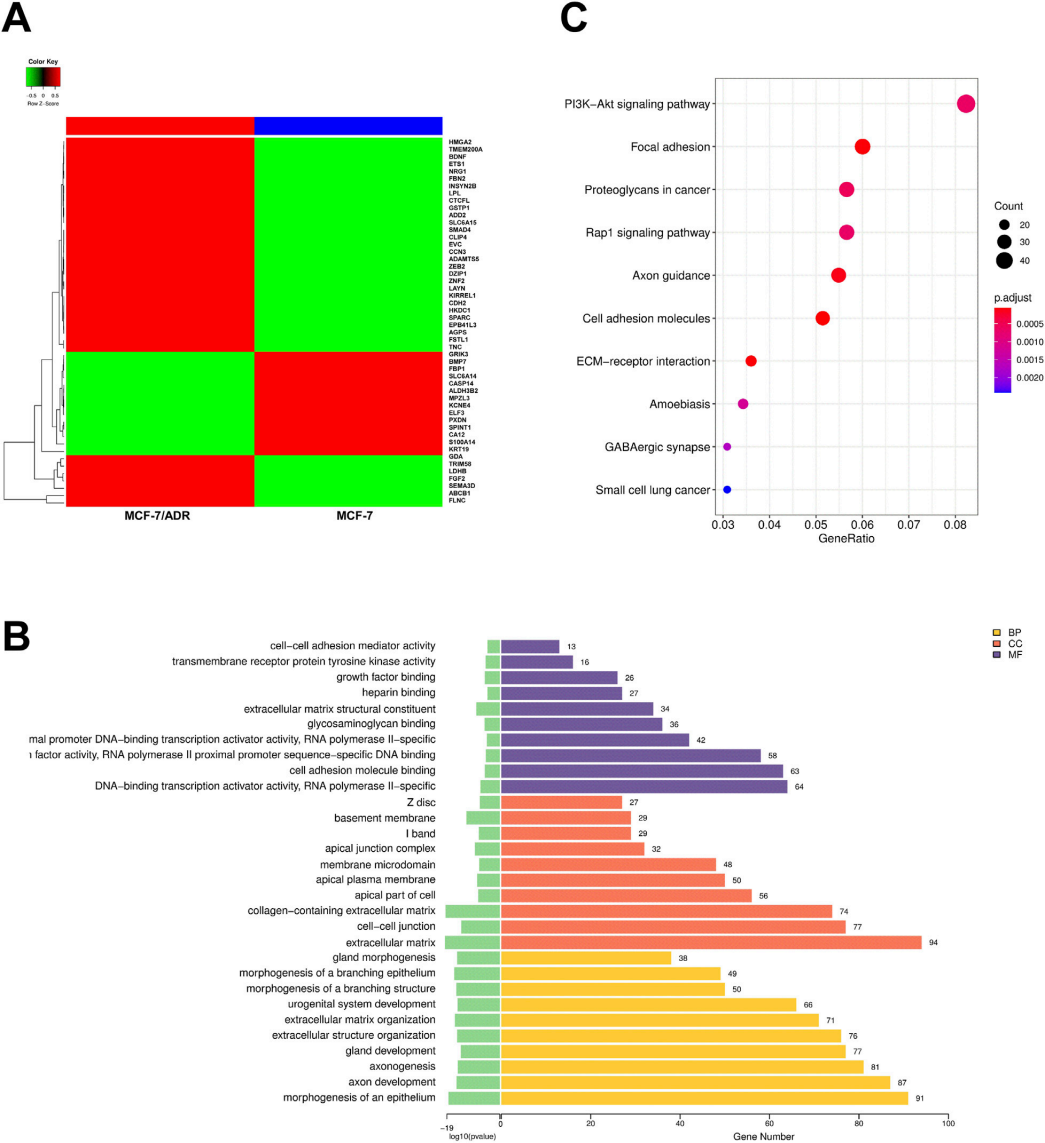
Autophagy is associated with chemotherapy resistance in breast cancer cells. **(A)** A heatmap shows the RNA sequencing analysis of MCF-7 and MCF-7/ADR cells with DE mRNAs. The gene signatures (top 50) are indicated in the right panel. **(B)** GO enrichment analysis (top 10) of DE mRNAs. Yellow represents biological process (BP), red represents cellular component (CC), and purple represents molecular function (MF). **(C)** KEGG pathway analysis (top 10) of DE mRNAs.

Autophagy has been demonstrated to be a potential mechanism that may promote chemotherapy resistance. Based on bioinformatics analysis, we hypothesized that BRMS1L enhances chemotherapy sensitivity by regulating autophagy. We performed RNA sequencing (RNA-seq) on MCF-7 and MCF-7/ADR cells, from which 1,431 differentially expressed mRNAs (DE mRNAs) were identified through the differential expression analysis (Figure 3A). To explore the potential effect of these DE mRNAs, we carried out Gene Ontology (GO) categories and Kyoto Encyclopedia of Genes and Genomes (KEGG) analysis. The GO analysis included the biological process (BP), cellular component (CC), and molecular function (MF). Among them, the BP analysis indicated that the DE mRNAs could regulate the morphogenesis of an epithelium, axon development, and so forth. The CC analysis revealed that they were enriched in the extracellular matrix, cell–cell junctions, and so forth. MF analysis identified that they were related to DNA-binding transcription activator activity, RNA polymerase II-specific functions, and so forth (Figure 3B). The KEGG pathway analysis revealed that these DE mRNAs might participate in the PI3K−Akt signaling pathway, Rap1 signaling pathway, and so forth (Figure 3C). It is worth mentioning that the PI3K–Akt signaling pathway attracts our attention because of its tightly association with the occurrence of autophagy (Shari et al., 2023; Zhang et al., 2022). The results suggested that autophagy would occur on MCF-7 and MCF-7/ADR cells; however, it remains unclear whether autophagy also occurs under the influence of BRMS1L on breast cancer cells. To identify our hypothesis, we performed the following experiments.

**FIGURE 4:**
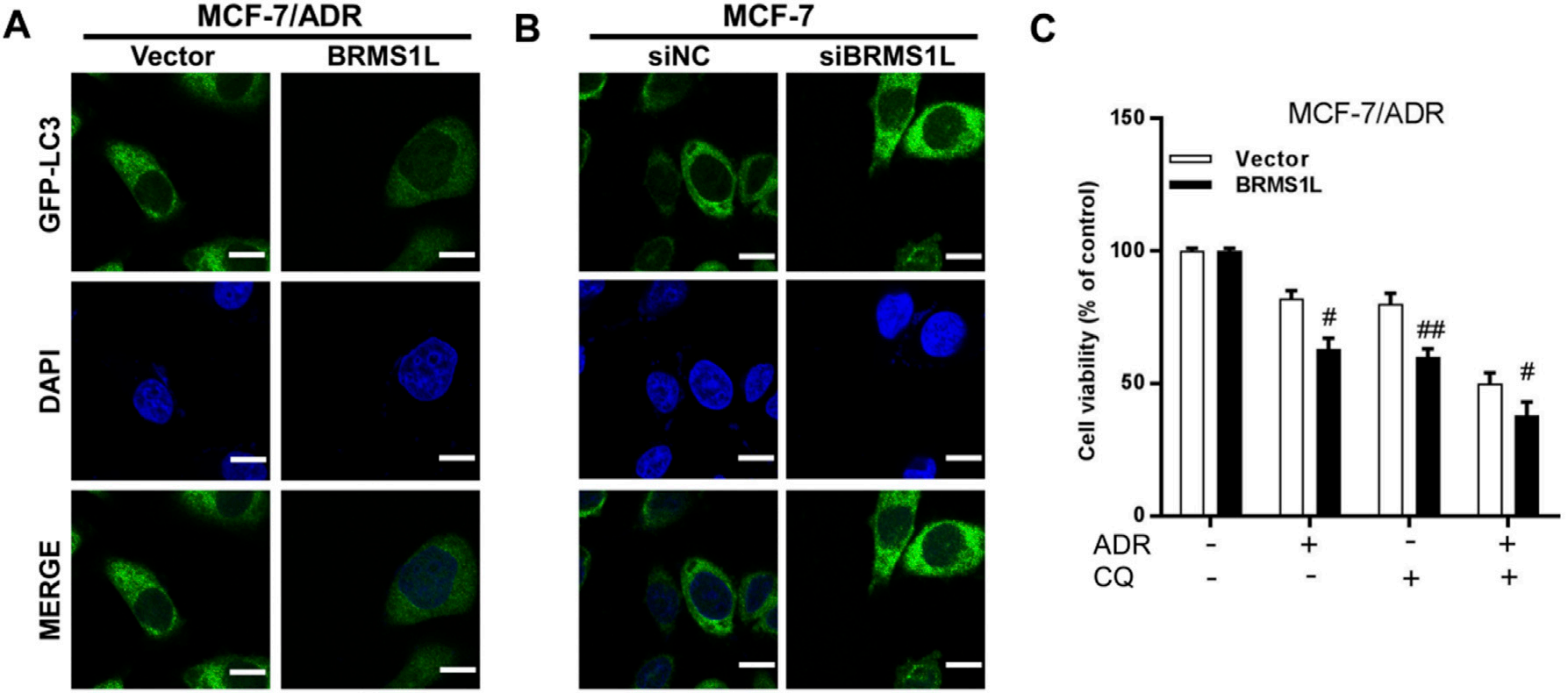
BRMS1L inhibits Adriamycin-induced autophagy in breast cancer cells. **(A,B)** Immunofluorescent staining of LC3 in MCF-7/ADR and MCF-7 cells. **(C)** Cell viability was measured with or without treatment of Adriamycin or CQ in MCF-7/ADR cells. BRMS1L vs. vector, ###, *p* < 0.001; #, *p* < 0.05.

LC3 (microtubule-associated protein 1 light chain 3), one of the known autophagy markers, was commonly used to examine the autophagy levels. LC3-I is located in the cytoplasm, and it can conjugate with phosphatidylethanolamine to form LC3-II and migrate to autophagosome membranes. To explore the relationship between autophagy and chemotherapy sensitivity, we transfected the cells with GFP–LC3 plasmid to visualize the autophagy after silencing or enforcing BRMS1L, and we found that following ectopic BRMS1L infection, GFP–LC3 was reduced in MCF/ADR cells. Conversely, GFP–LC3 was significantly upregulated in MCF-7 cells after silencing BRMS1L (Figures 4A,B). Next, whether the inhibition of autophagy was associated with BRMS1L-mediated enhanced ADM sensitivity was further investigated. Chloroquine (CQ), a lysosomotropic agent, is reported to be efficient at inhibiting autophagy by preventing the fusion of lysosomal and autophagosome. Cell viability assays demonstrated that although the ectopic expression of BRMSL enhanced sensitivity to chemotherapy, their sensitivity was restored by cotreatment with CQ (Figure 4C). Taken together, these results demonstrate that BRMS1L increases chemotherapy sensitivity via the inhibition of ADM-induced autophagy.

### BRMS1L inhibits autophagy via downregulation of ATG5

**FIGURE 5:**
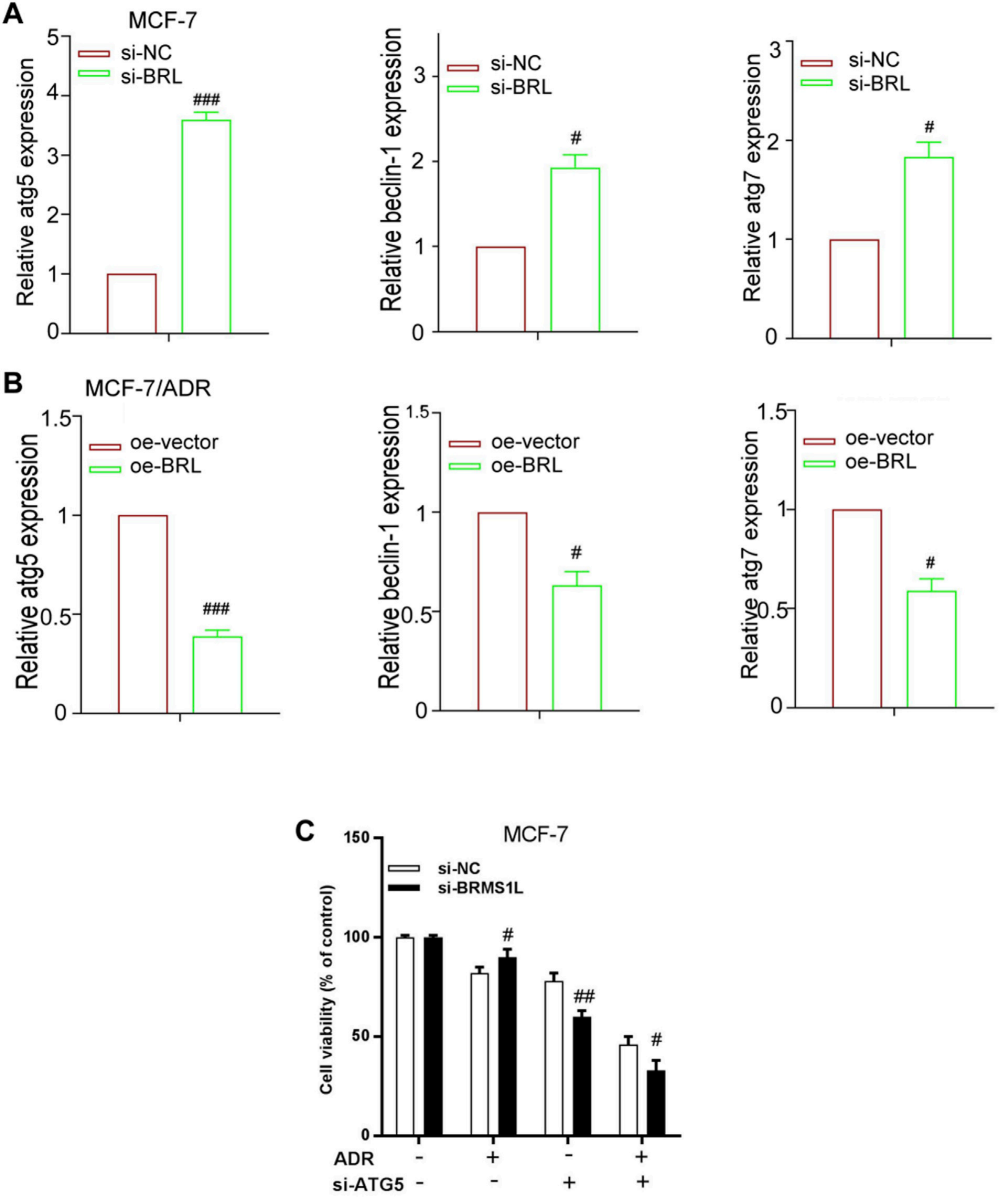
BRMS1L inhibits autophagy via downregulation of ATG5. **(A,B)** Expression levels of ATG5, ATG7, and Beclin-1 were measured using qRT-PCR in MCF-7/ADR and MCF-7 cells that were treated with the pcDNA3 vector carrying BRMS1L or si-BRMS1L, along with their controls. **(C)** Cell viability was determined in MCF-7 cells that were co-transfected with BRMS1L-siRNA and ATG5-siRNA. Si-BRMS1L vs. si-NC, oe-vector vs. oe-BRMS1L, ###, *p* < 0.001; ##, *p* < 0.01; #, *p* < 0.05. si-NC, negative control; si-BRMS1L, BRMS1L-siRNA; oe-vector, vector; oe-BRMS1L, BRMS1L; si-ATG5, ATG5-siRNA.

To investigate the mechanism by which BRMS1L inhibited ADM-induced protective autophagy in breast cancer cells, the mRNA levels of several autophagy-related genes were examined. As shown in Figures 5A,B, silencing BRMS1L increased the mRNA levels of ATG5, Beclin-1, and ATG7, whereas the overexpression of BRMS1L in MCF-7/ADR cells reduced the mRNA level of ATG5, Beclin-1, and ATG7. As the ATG5 mRNA level presented the most prominent alternation, we focused on ATG5 in the subsequent experiments. ATG5, a part of the lipid kinase complex, could induce to form the initial stages of autophagosome and served as the autophagy marker. In this section, we investigate whether BRMS1L-mediated ATG5 downregulation contributes to the inhibition of autophagy in breast cancer cells. According to the cell viability assays, the MCF-7 cells demonstrate that co-transfection with ATG5-siRNA efficiently alleviated the promoting effect of BMRS1L-siRNA (Figure 5C). Collectively, these results indicated that BRMS1L suppresses ATG5 expression, which inhibits the ADM-induced protective autophagy in breast cancer cells.

### BRMS1L enhances chemotherapy sensitivity *in vivo*

**FIGURE 6:**
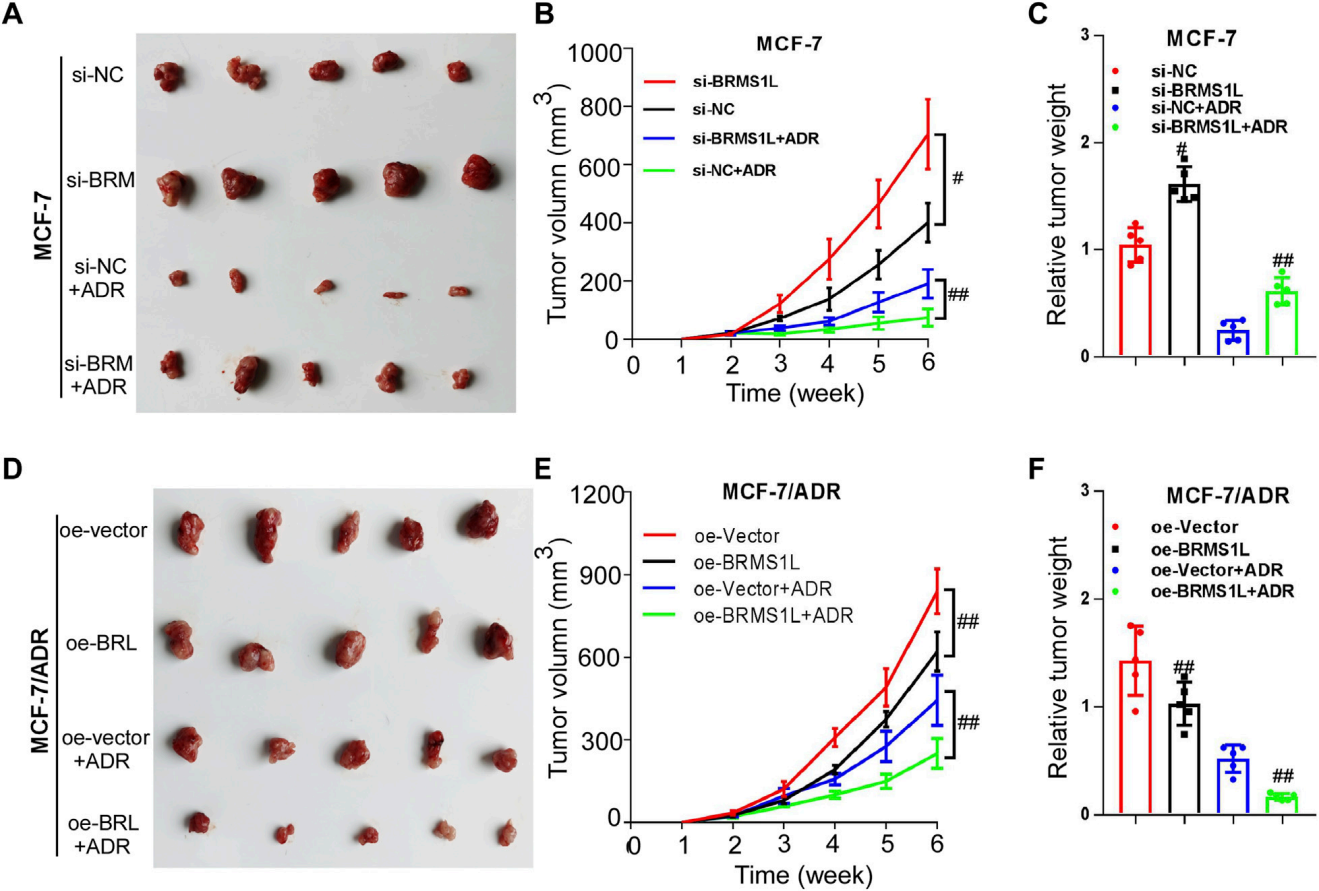
BRMS1L enhances chemotherapy sensitivity *in vivo*. **(A–C)** Silencing BRMS1L dramatically promoted the growth of breast xenograft tumors upon Adriamycin treatment. **(D–F)** Overexpression of BRMS1L significantly inhibited the growth of breast cancer xenograft tumors upon Adriamycin treatment. Si-BRMS1L vs. si-NC, oe-vector vs. oe-BRMS1L, Si-BRMS1L+ADR vs. si-NC+ADR, oe-vector+ADR vs. oe-BRMS1L+ADR; ##, *p* < 0.01; #, *p* < 0.05.

To further investigate whether BRMS1L enhances chemotherapy sensitivity *in vivo*, we established xenograft models using MCF-7 and MCF-7/ADR cells. As shown in Figure 6, silencing BRMS1L dramatically promoted the growth of breast xenograft tumors in nude mice upon ADM treatment (Figures 6A–C; Supplementary Figure S2). However, overexpression of BRMS1L significantly inhibited the growth of breast cancer xenograft tumors upon ADM treatment (Figures 6D–F). These results indicated that BRMS1L increased the antiproliferative effects of chemotherapy *in vivo*.

## Discussion

Although neoadjuvant chemotherapy has significantly improved the pathological complete response (PCR) rate in breast cancer patients, chemotherapy resistance remains a major obstacle to successful cancer therapy. In recent years, numerous clinical trials have reported a PCR rate of up to 60% in HER-2-positive breast cancer and triple-negative breast cancer (TNBC), which is far higher than that in luminal breast cancer (Shi et al., 2024; Gianni et al., 2016). In reality, luminal breast cancer, as the most common breast cancer subtype, often has a lower pathological grade, lower relapse rate, and better chemotherapy response. However, approximately 10% patients could gain benefit from NACT (Collins et al., 2021; Hu et al., 2019; Yu et al., 2014). Therefore, it is worthy to focus on increasing the PCR rate of luminal breast cancer at the moment. To investigate more new treatment strategies and enhance their clinical relevance, the representative breast cancer cells MCF-7 and ADM-resistant MCF-7 (MCF/ADR) were highlighted in our study.

At first, we demonstrated that reduced BRMS1L expression correlates with poor response to neoadjuvant chemotherapy and a poor prognosis. BRMS1L is one of the components in the Sin3A/HDAC complex. Our previous study demonstrated that BRMS1L suppresses invasion and metastasis of breast cancer cells by inhibiting EMT. The biological effect of BRMS1L is mediated by the epigenetic silencing of the *FZD10* gene through the recruitment of HDAC1 to its promoter and enhancing histone H3K9 deacetylation (Gong et al., 2014). Furthermore, BRMS1L expression in breast cancer cells is associated with less metastasis and better clinical outcome. Previous studies revealed the tumor suppressor role of BRMS1L in several types of malignancies. For example, BRMS1L suppresses metastasis by inhibiting the β-catenin/wnt pathway in ovarian cancer (Cao et al., 2018). Koyama et al. (2017) found that BRMS1L is one of the mediators downstream of the p53 pathway and inhibits brain cancer invasion and migration. Zhou et al. (2020) revealed that BRMS1L exerted their metastasis-suppressing role by transcriptionally repressing the ITGA7 expression in esophageal squamous cell carcinoma. Recently, Cao et al. (2024) found that the knockdown of BRMS1L expression was correlated with sensitivities to cisplatin-based chemotherapy and conferred anticancer activity in non-small-cell lung cancer by transcriptionally inducing a redox imbalance in the GPX2–ROS pathway. These studies suggest that BRMS1L could be a therapeutic target for cancer. However, there are still limited research on breast cancer about BRMS1L, which implicated that in-depth mechanism on carcinogenesis should be explored. In the present study, we were interested in the role of BRMS1L in regulating chemotherapy sensitivity in breast cancer cells.

ADM results in DNA damage, prevents DNA repair, and leads to cell apoptosis or inhibits the activation of topoisomerase to cause cell death (Yang et al., 2014; Zhang et al., 2012). The regulation of BRMS1L by chemotherapy in breast cancer cells remains undisclosed; we therefore carried out experiments and demonstrated that chemotherapy-resistant breast cancer cells exhibited decreased BRMS1L expression. Furthermore, the crucial problem we are concerned with is the molecular mechanism by which BRMS1L regulates chemotherapy sensitivity. So far, autophagy is considered to be the most important stress regulatory machinery responsible for drug administration (Gewirtz, 2014; Aydinlik et al., 2017; Park et al., 2016; Guo et al., 2016; Chittaranjan et al., 2014). Although the controversy over the prosurvival or anticancer effect of autophagy remains heated, data from *in vitro* and *in vivo* studies seem to support the hypothesis that autophagy facilitates resistance to chemotherapy treatment. Thus, the inhibition of autophagy may facilitate the re-sensitivity of therapeutic-resistant cancer cells to anticancer drugs. Many studies demonstrated that autophagy inhibitors could chemosensitize cancer treatment, which has been considered a novel strategy to enhance chemotherapy sensitivity (Cuomo et al., 2019; Kuusisto et al., 2001; Tanida et al., 2005; Yu et al., 2017; Lapierre et al., 2015). For this study, we performed RNA-seq and found that the differential genes in chemoresistant cells were relevant to autophagy-related signaling pathways, such as the PI3K−Akt signaling pathway and the Rap1 signaling pathway. It has been reported that the inhibition of the PI3K/AKT/NF-κB pathway could induce autophagy in resistant breast cancer cells (Shari et al., 2023). In addition, 6-MDS could induce autophagy of MCF-7 cells by suppressing the PI3K/AKT/mTOR signaling pathway (Zhang et al., 2022). Based on previous studies, we wonder whether BRMS1L is capable of promoting chemotherapy sensitivity via regulating autophagy. The elevated autophagy level in chemotherapy-resistant cells was validated first. We further investigated the effects of BRMS1L on autophagy and found that the inhibition of autophagy was associated with BRMS1L-mediated enhanced chemosensitivity. Interestingly, as various mechanisms of chemotherapy resistance were identified, the abovementioned reaction was found to occur not only with ADM chemotherapy but also with other different chemotherapy drugs such as paclitaxel and cisplatin, presenting as cross-resistance in breast cancer (R et al., 2022; Cocco et al., 2022; Cheng et al., 2022; Zhang et al., 2025). Thus, we demonstrated that BRMS1L significantly enhanced chemotherapy sensitivity via inhibiting protective autophagy in breast cancer cells.

Several conserved autophagy-related genes are involved in autophagy, and these genes have multiple functions in various physiological contexts. Among these genes, the ATG5 protein in a conjugated form with ATG12 and ATG8 (LC3) is involved in the early stages of autophagosome formation and plays an important role in the maturation of autophagosomes (Mizushima, 2020). Accumulating evidence demonstrated that ATG5 has a significant impact on autophagy that leads to chemoresistance in various tumor cells. For instance, upregulation of ATG5 depresses the sensitivity of prostate and lung cancer cells to chemotherapy by inducing autophagy (Cristofani et al., 2018; Wang et al., 2022), whereas silencing of ATG5 suppresses autophagy and increases the sensitivity to imatinib mesylate (Tong et al., 2012). Moreover, recent research demonstrated that ATG5 is elevated by an early growth response factor, triggering autophagy and promoting radioresistance of HCC cells (Peng et al., 2017). In this study, we, for the first time, demonstrated that BRMS1L enhanced chemotherapy sensitivity by inhibiting ATG5. Our findings suggested a novel role of BRMS1L in chemosensitivity.

In this study, we demonstrated that BRMS1L enhanced the chemotherapy sensitivity and subsequently promoted the prognosis of breast cancer patients by inhibiting autophagy. However, our study had several limitations. First, only ADM was used in our study. We did not validate whether BRMS1L could modulate chemoresistance induced by the other chemodrugs, such as paclitaxel and cisplatin. Second, only one chemoresistant cell line was constructed in our study. Third, deferentially expressed genes in MCF7/ADM vs. MCF7 cells were not validated. Therefore, further investigation should be continued to better understand the impact of BRMS1L on chemosensitivity. In summary, our discovery will not only increase our knowledge on the role of BRMS1L in breast cancer but also provide a novel biomarker for developing the sensitizing strategy and predicting response to breast cancer chemotherapy.

## Methods

### Cell culture and treatment

The human breast cancer cell line MCF-7 was purchased from Cell Bank (Chinese Academy of Sciences). The ADM-resistant cell line, MCF-7/ADR, has long been cultured in our laboratory and was treated with a low concentration of ADM (1 μM) every 4 weeks to maintain cell resistance (Supplementary Figure S1). Both the MCF-7 and MCF-7/ADR cells were grown in DMEM (Gibco, United States) supplemented with 10% fetal bovine serum (Gibco, United States) and antibiotics (100 U/mL penicillin and 100 mg/mL streptomycin) (Gibco, United States) at 37 °C with 5% CO_2_.

For transfection assays, siRNA duplexes targeting BRMS1L were designed and synthesized by RiboBio (Guangzhou, China). A BRMS1L overexpression vector was constructed from pcDNA3 (Invitrogen, United States). When cells were grown overnight to 50%–70% confluency (Gong et al., 2014), Lipofectamine 2000 (Invitrogen, United States) was used for transient transfection of siRNA and vectors (Supplementary Table S5). The transfection efficiency was examined using Western blot analysis after the transfected cells were incubated for further 48 h (Supplementary data).

For CCK-8 assays, cells were cultured in 96-well culture plates (5 × 10^3^/well). Following adhesion, the cells were treated with different concentrations of ADM (Sigma-Aldrich, st. Louis, MO, United States) for 48 h, and drug sensitivity was confirmed using the CCK-8 assay (Dojindo, Kumamoto, Japan). The culture medium was discarded after incubation, and 10 μL of CCK‐8 reagent was added to each well. The spectrophotometric absorbance of the cells was measured using an Ultra Multifunctional Microplate Reader (Tecan, Durham, NC, United States) at 450 nm. Cell viability and the half-maximal inhibitory concentration (IC50) of ADM were calculated according to the OD value. Experiments were conducted at least in triplicate using separate cultures.

### Clinical samples

Fresh breast cancer biopsy tissues for examining BRMS1L expression were obtained from 138 patients with breast cancer, who were treated with NACT. All the patients were female, aged ≥18 years, with newly diagnosed, previously untreated, and pathological confirmed invasive breast cancer. Other key inclusion criteria included normal liver, renal, and bone marrow functions. For chemotherapy, the key exclusion criteria included stage IV breast cancer and contraindications. The patients were grouped as the chemosensitive group and the chemoresistant group based on the neoadjuvant chemotherapy response (RECIST 1.1 criteria). All tissue samples were collected from the First Affiliated Hospital of Jinan University between 2017 and 2020, and all patients in the study signed informed consents. All experimental procedures were approved by the Ethics Committee of the First Affiliated Hospital of Jinan University (Guangzhou, China).

### Immunohistochemistry

IHC was performed on paraffin sections of breast tissues according to the standard LSAB protocol (DAKO, Glostrup, Denmark), using primary antibodies against BRMS1L (1:200, NBP2-14362, Novus, Centennial, CO, United states). The BRMS1L expression level was scored semi-quantitatively using the IRS ¼ SI (staining intensity) x PP (percentage of positive cells) as described. In brief, SI was determined as follows: 0, negative; 1, weak; 2, moderate; and 3, strong. PP was defined as follows: 0, <1%; 1, 1%–10%; 2, 11%–50%; 3, 51%–80%; and 4, >80% positive cells. Ten visual fields from different areas of each tumor were used for the IRS evaluation. IRS ≤4 was defined as low BRMS1L expression and IRS >4 was defined as high BRMS1L expression.

### RNA isolation and quantitative real-time polymerase chain reaction

Total RNA was extracted from breast cancer tissue samples and cell lines using TRIzol (Invitrogen). Complementary DNA synthesis was performed using PrimeScript reverse transcription reagents (Takara, Shiga, Japan, R060A). Quantitative PCR (qPCR) was carried out on LightCycler480 (Roche, Germany) using SYBR Premix EX Taq reagent (Takara, Shiga, Japan, RR420A). The expression of target genes was calculated using the 2^−ΔΔ^CT (cycle threshold) method compared with the house keeping gene *GAPDH*. All experiments were repeated thrice. The primer sequences for BRMS1L, ATG5, ATG7, Beclin1, and GAPDH are listed in Supplementary Table S3.

### Identification of differentially expressed mRNAs

A total amount of 1 µg RNA per sample from cells was used as the input material for the RNA sample preparations. Sequencing libraries were generated using the NEBNext^®^ UltraTM RNA Library Prep Kit for Illumina^®^ (CatalogE7530L, NEB, United States) following the manufacturer’s recommendations (GSE306386). Then, the libraries were pooled and sequenced on an Illumina NovaSe™ 6000 platform and finally generated 150 bp paired-end reads.

For quality control, clean reads were obtained from removing reads containing adapter, reads containing ploy-N, and low-quality reads from raw data (raw reads) of fastq format. At the same time, Q20, Q30, and GC contents of clean data were calculated. All the downstream analyses were based on the clean data with high quality. Reference genome and gene annotation files were downloaded from the genome website. Hisat2 (v2.0.5) was selected as the mapping tool because Hisat2 can generate a database of splice junctions based on the gene model annotation file. The mapped reads of each sample were assembled using StringTie with default parameters. Then, all transcripts were merged to reconstruct a comprehensive transcriptome using gffcompare software.

For quantification of the gene expression level, RSEM was used to count the read numbers mapped to each gene. Fragments per kilobases per million reads (FPKM) and transcripts per million reads (TPM) were calculated to estimate the mRNA expression levels. For differential expression analysis (DEA), the DESeq2 (v1.34.0) R package was used to identify differential genes between MCF-7 and MCF-7/ADR cells. The *p*-values were adjusted using the Benjamini and Hochberg method to control the false discovery rate (FDR). A corrected *p*-value of 0.05 (adjusted *p*-value <0.05) and an absolute fold change of 2 (|log2FC| >1) were set as the thresholds for significant differential expression. Detailed RNA sequencing data are shown in Supplementary Table S4, and the PCA plot is shown in Supplementary Figure S3.

### GO categories and KEGG enrichment analysis

The relevant analysis of DE mRNAs was performed using the clusterProfiler package in R version 3.6.0 (Vienna, Austria). The raw *p*-values of both GO and KEGG enrichment analyses were corrected using the Benjamini–Hochberg method. The significant GO terms and KEGG pathways were constructed when the enriched gene count ≥2 and the significance threshold-adjusted *p*-value *<*0.05.

### Tumor xenografts

Female BALB/c nude mice (6 weeks of age, specific pathogen-free [SPF] grade) were used to establish subcutaneous xenograft models. MCF-7 and MCF-7/ADR cells (1 × 10^6^) were subcutaneously injected into the fat pad. BRMS1L-targeting small interfering RNA (si-BRMS1L) and the control, along with BRMS1L-overexpressing plasmid (pcDNA-BRMS1L) and the control vector, were dissolved in sterile normal saline and injected via the tail vein every 3 days. When the tumor volumes were approximately 100–200 mm^3^, the mice were administered doxorubicin (5 mg/kg, 0.5 mg/mL, Sigma) via tail vein injection every 7 days. The growth of tumors and the body weights of mice were monitored after 6 weeks (TV = length × width^2^ × 0.5). Then, the mice were sacrificed by carbon dioxide inhalation, as suggested by the National Institutes of Health (NIH) guidelines for the euthanasia of rodents, following which the tumors were dissected and weighed. The experimental protocol was approved by the Animal Research and Care Committee of Jinan University. All methods performed on animals were reported in accordance to the ARRIVE guidelines.

### Statistics

All statistical analyses were performed using SPSS for Windows version 20.0. Student’s t-test was used for the comparison of two independent groups. The χ^2^ test was applied to analyze the association between the BRMS1L expression level and clinicopathological status. The Kaplan–Meier survival curves of disease-free survival (DFS) and overall survival (OS) were plotted with a median follow-up of 45 months. The log-rank test was used to analyze survival differences. All experiments *in vitro* were performed independently for at least three times and in triplicate for each time. All results are expressed as mean ± s.d, and mean values of three experiments are shown. A *p*-value of 0.05 was considered statistically significant in all cases.

## Data Availability

The original contributions presented in the study are publicly available. This data can be found in the Gene Expression Omnibus (GEO) repository with the accession number GSE306386.

## Abbreviations

BRMS1L, BReast cancer Metastasis Suppressor 1 Like; HDAC, Histone DeACetylase; IHC, ImmunoHistoChemistry; NACT, NeoAdjuvant ChemoTherapy; ADM, ADriaMycin.
