# Supplementary material for: BRMS1L promotes chemotherapy sensitivity by inhibiting autophagy in breast cancer
Source: Front Genet. 2025 Nov 7;16:1670001. doi: 10.3389/fgene.2025.1670001 (PMC12634030; doi:10.3389/fgene.2025.1670001)
Supplement: Supplementary file 1 [file DataSheet1.docx]

**Supplementary Table 1. Clinical response to neoadjuvant chemotherapy in patients with high or low BRMS1L expression No.(%)**

| Response to chemotherapy | Total No. | BRMS1L expression | | χ 2 | *p* |
| --- | --- | --- | --- | --- | --- |
|  |  | High（IRS＞4） | Low（IRS≤4） |  |  |
| CR | 23 | 15 | 8 |  |  |
| PR | 60 | 34 | 26 |  |  |
| SD | 27 | 13 | 14 |  |  |
| PD | 28 | 8 | 20 |  |  |
| Total | 138 | 70 | 68 | 8.35 | 0.039 |

CR: Complete Response; PR: Partial Response; SD: Stable Disease; PD: Progressed Disease. χ 2 test were used to calculate p-values.

**Supplementary Table 2. Treatment paradigms between patients with high and low BRMS1L expression No.(%)**

| Response to chemotherapy | Total No. | BRMS1L expression | | P |
| --- | --- | --- | --- | --- |
|  |  | High（IRS＞4） | Low（IRS≤4） |  |
| Neoadjuvant chemotherapy | | |  | 0.111 |
| CE-T/CET | 69 | 37 | 32 |  |
| EC-TH | 28 | 15 | 13 |  |
| TCbH | 21 | 10 | 11 |  |
| Other | 20 | 8 | 12 |  |
| Surgical therapy | |  |  | 0.267 |
| Mastectomy | 97 | 46 | 51 |  |
| Conservation | 41 | 24 | 17 |  |
| Cycles of chemotherapy | | |  | 0.766 |
| four | 22 | 11 | 11 |  |
| six | 51 | 24 | 27 |  |
| eight | 65 | 35 | 30 |  |
| Endocrine therapy | |  |  | 0.552 |
| AI | 48 | 23 | 25 |  |
| TAM | 33 | 15 | 18 |  |
| None | 57 | 32 | 25 |  |

C: cyclophosphamide; E: epirubicin; T: docetaxel; H: trastuzumab; Cb: carboplatin

χ 2 test were used to calculate *p*-values.

**Supplementary Table 3. The primer sequences for target genes**

| Target genes | Primer sequences |
| --- | --- |
| BRMS1L | Forward  5’-AGTGAAAACGGAACCACCTG-3’  Reverse  5’-TATGGACCTGAAACAACAACTGG-3’ |
| ATG5 | Forward  5’-TGGGCCATCAATCGGAAACTC-3’  Reverse  5’- TGCAGCCACAGGACGAAACAG-3’ |
| ATG7 | Forward  5’-GCGGAATTCCATGGCGGCAGCTACGG-3’  Reverse  5’-ACGCGGCCGCTCAGATGGTCTCATCAT-3’ |
| Beclin1 | Forward  5’-TGAGGGATGGAAGGGTCTAAG-3’  Reverse  5’-CCTAAGGAAAAAAGTCATTTGTTAT-3’ |
| GAPDH | Forward  5’-GCTGGCGCTGAGTACGTCGTGGAGT-3’  Reverse  5’-CACAGTCTTCTGGGTGGCAGTGATGG-3’ |

**Supplementary Table 4. RNA sequencing data obtained in the study**

| **Sample** | **Raw**  **Reads** | **Raw**  **Bases** | **rRNA** | **Clean**  **Reads** | **Clean**  **Bases** | **Clean**  **Ratio** | **Q20** | **Q30** | **GC** |
| --- | --- | --- | --- | --- | --- | --- | --- | --- | --- |
| MCF-7 | 154805218 | 22724206572 | 78535110  (50.73%) | 74597532 | 10942139560 | 98.26% | 97.42% | 93.18% | 45.07% |
| MCF-7/ADR | 136518522 | 20121576297 | 64137822  (46.98%) | 70749444 | 10424410689 | 98.20% | 97.39% | 93.14% | 43.49% |

**Supplementary Table 5. siRNA sequences**

| BRMS1L-siRNA | 5’-GAAGACUGGACAACAAUUATT-3’  5’-UAAUUGUUGUCCAGUCUUCTT-3’ |
| --- | --- |

**Western blotting**


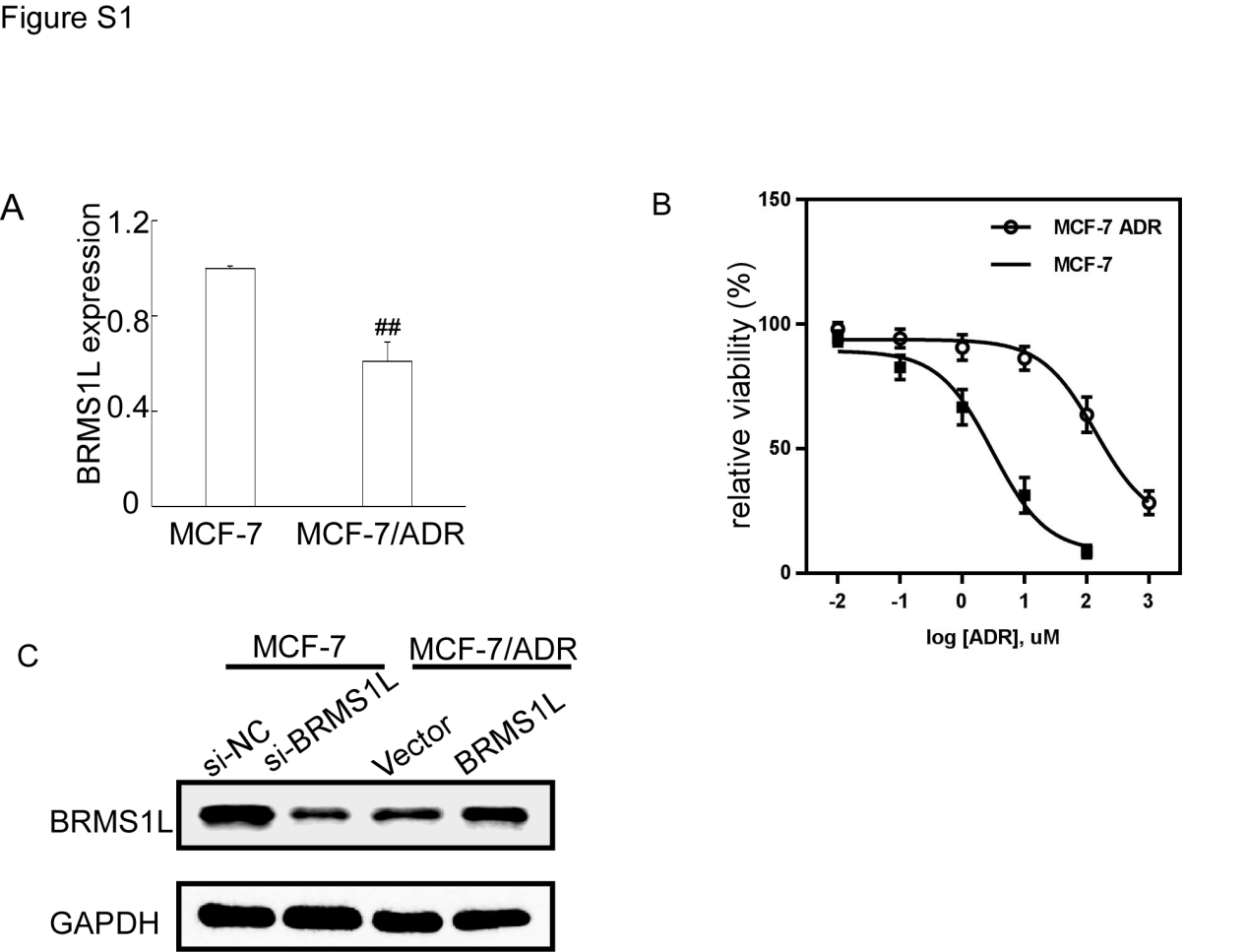
Protein extracts were resolved in 8%-15% SDS-PAGE, transferred to PVDF membranes, and probed with antibodies against BRMS1L (NBP2-14362, NOVUS, 1:1000) and β-actin (A3854, Sigma, 1:1000). Peroxidase-conjugated secondary antibodies against rabbit (A0545, Sigma, 1:1000) was used, and the antigen-antibody reaction was visualized by the enhanced chemiluminescence assay (WBKLS0500, ECL, Millipore).

Figure S1. A. BRMS1L expression in MCF-7 and ADR cells. B. Relative viability in MCF-7 and ADR cells. C. BRMS1L expression in cells. MCF-7/ADR vs. MCF7, ##, *p*<0.01.


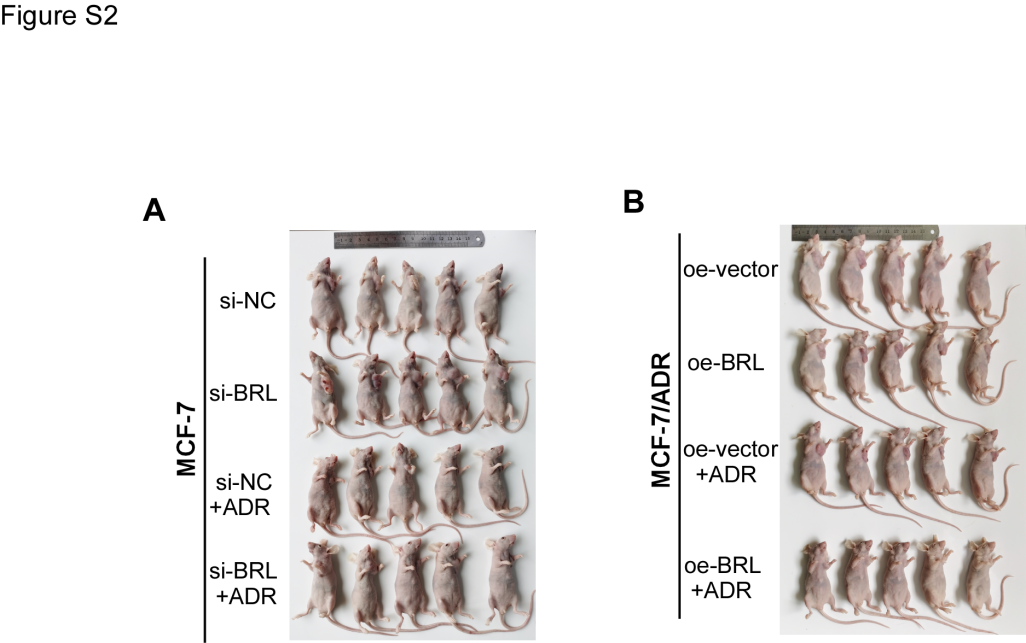


Figure S2. A&B Growth of breast xenograft tumors upon knockdown and overexpression of BRMS1L treatment. BRL, BRMS1L.

Figure S3


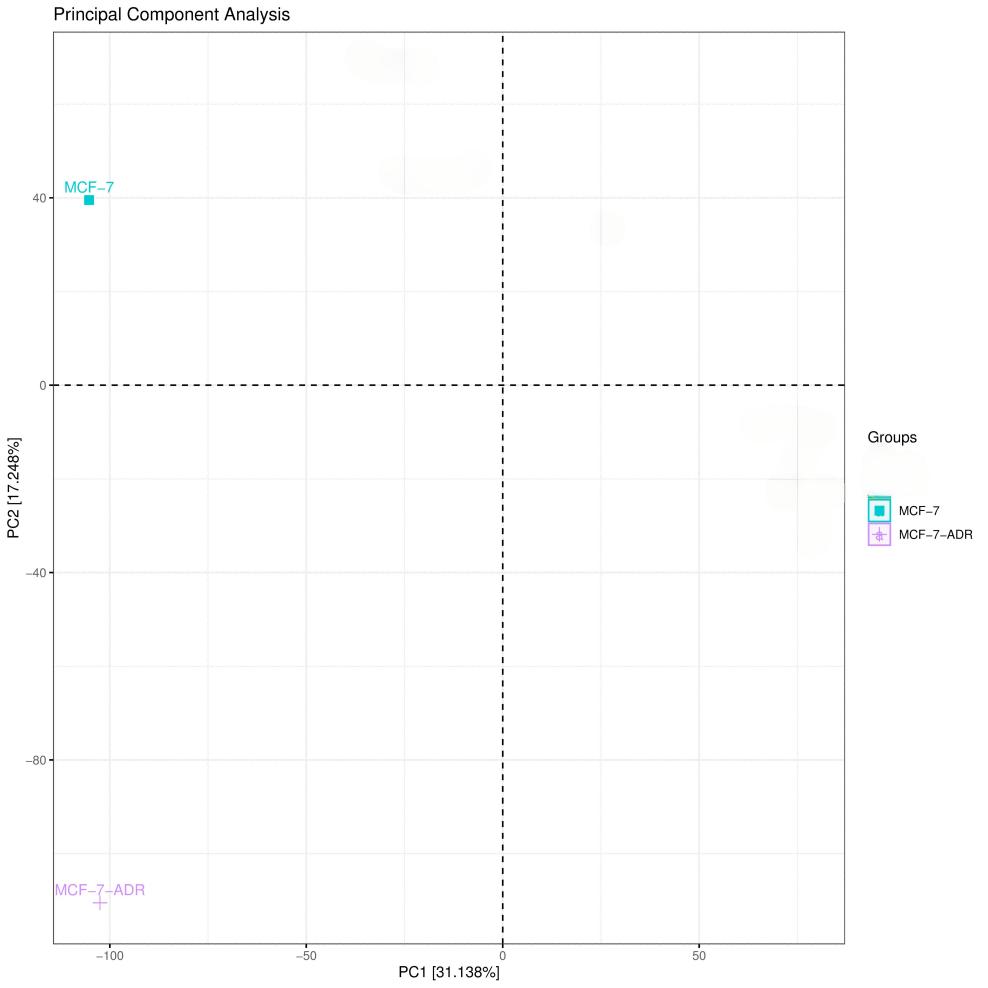


Figure S3. The PCA plot between MCF-7 and MCF-7/ADR groups.
